# The paradoxical effect of COVID-19 outbreak on loneliness

**DOI:** 10.1192/bjo.2020.163

**Published:** 2021-01-11

**Authors:** David Bartrés-Faz, Dídac Macià, Gabriele Cattaneo, Roger Borràs, Clara Tarrero, Javier Solana, José M. Tormos, Alvaro Pascual-Leone

**Affiliations:** Department of Medicine, Faculty of Medicine and Health Sciences, University of Barcelona, Spain; and Guttmann Institute, University Institute of Neurorehabilitation affiliated to the Autonomous University of Barcelona (UAB), Spain; Department of Medicine, Faculty of Medicine and Health Sciences, University of Barcelona, Spain; Guttmann Institute, University Institute of Neurorehabilitation affiliated to the Autonomous University of Barcelona (UAB), Spain; and Autonomous University of Barcelona, Spain; Department of Child and Adolescent Psychiatry and Psychology, Institute of Cllinical Neurosciences, Clinical and Provincial Hospital of Barcelona, Spain; Guttmann Institute, University Institute of Neurorehabilitation affiliated to the Autonomous University of Barcelona (UAB), Spain; and Autonomous University of Barcelona, Spain; Guttmann Institute, University Institute of Neurorehabilitation affiliated to the Autonomous University of Barcelona (UAB), Spain; and Autonomous University of Barcelona, Spain; Guttmann Institute, University Institute of Neurorehabilitation affiliated to the Autonomous University of Barcelona (UAB), Spain; and Autonomous University of Barcelona, Spain; Guttmann Institute, University Institute of Neurorehabilitation affiliated to the Autonomous University of Barcelona (UAB), Spain; and Hinda and Arthur Marcus Institute for Aging Research, Hebrew SeniorLife, Harvard Medical School, Boston, Massachusetts, USA.

**Keywords:** Psychological testing, rating scales, social functioning, community mental health teams, psychosocial interventions

## Abstract

As in previous periods of quarantine, lockdown confinement measures dictated to control SARS-CoV-2 would be expected to negatively affect mental health. We investigated the immediate effects (over a 10 day period) of a strict nationwide stay-at-home order imposed in Spain, one of the countries most affected by the COVID-19 pandemic. Focusing our analysis on the feelings of loneliness, we obtained our measures within a social context characterised by strong and continuous public and governmental support for increasing social bonds and cooperation in order to face the common public threat. Leveraging data from the Barcelona Brain Health Initiative, a prospective population-based study cohort, the short UCLA Loneliness Scale was administered to 1604 participants 2 years and 1 year before the stay-at-home lockdown and repeated, on average, 10 days after the official confinement order issued by the Spanish government. Ratings of loneliness remained stable during the 2 years before lockdown; however, they decreased significantly during the early stages of home confinement. This effect was particularly significant for the item ‘feeling excluded from others’ and was also observed among individuals who were confined alone. Overall, the results suggest that gestures and manifestations of appreciation by people for the labour and efforts of certain individuals, along with official campaigns designed to promote feelings of inclusion and belonging, may have beneficial effects on feelings of loneliness, a negative emotional state strongly regarded as a risk factor for impaired mental and general health status. Further assessments during the later stages of home confinement are now warranted.

In early April 2020, almost 1.5 million cases of COVID-19 had been confirmed in more than 200 countries, and over 80 000 deaths had already been recorded. Spain had the second highest number of confirmed COVID-19 cases after the USA, and the highest rate of cases per inhabitant.^1^ As in many countries, the government in Spain issued a nationwide stay-at-home order imposing strict lockdown measures in mid-March.^2^

The potentially negative psychological effects of quarantine have been described elsewhere,^3^ including observations of the associations between loneliness and mental health issues (e.g. anxiety, depression, suicidal ideation) within the context of the COVID-19 pandemic,^4^ and of increases in feelings of loneliness following the stay-at-home restrictions in both general community samples^5^ and specific populations such as university students.^6^ However, across the world people have been coming together during the pandemic in new ways to cope with the crisis and the challenge of confinement. For example, in Spain, every evening at 20:00 h, from windows, front doors, balconies or terraces, thousands of people applauded frontline health professionals and acknowledged them as ‘heroes of the nation’. Families and friends found new ways to connect for online meals, drinks or games. As in other countries, the official motto of Spain's governmental COVID-19 campaign, ‘éste virus lo paramos unidos’ (united we will stop this virus) reinforced the importance of social bonds and cooperation.

In this study, we wanted to assess whether feelings of loneliness, defined as the feeling that one's desired quantity or quality of social connections is unfulfilled,^7,8^ in particular those referring to the subjective sense of social belonging, underwent changes during the early stages of the COVID-19 pandemic and, if so, whether they increased or decreased.

## Method

Study participants were part of the Barcelona Brain Health Initiative (BBHI, www.bbhi.cat/en), an ongoing longitudinal cohort study investigating the determinants of brain and mental health in middle and advanced age. The BBHI involves periodic medical, cognitive, brain imaging and biological assessments.^9^ The present study included a subsample of 1604 participants demographically representative of the full BBHI cohort (*n* = 5592 as of April 2020). The mean age of the subsample was 55.7 years (s.d. 7.3); 65% were women, and 75% had completed higher education, 22% secondary and 3% only primary. The corresponding figures for the full BBHI cohort were: mean age 54.4 years (s.d. 7.2), 67% women, 71% completed higher education, 25% secondary and 4% only primary.

None of the respondents reported a diagnosis of a psychiatric or neurological disorder by a physician, and all completed the three waves of loneliness assessment using the short UCLA Loneliness Scale,^10^ which consists of three items in which participants had to rate the frequency of several experiences (‘How often do you feel isolated from others?’, ‘How often do you feel excluded?’ and ‘How often do you feel that you lack company’) on a three-point Likert scale (options: ‘1: Hardly ever’, ‘2: Some of the time’, or ‘3: Often’). The scale was administered through the BBHI web platform (https://bbhi.cat/en/), a personalised platform where participants are requested to fill in a series of questionnaires that assess their self-perceived health and engagement in various lifestyles (cognitive, physical, sleep, social and nutrition habits). For the current study, UCLA ratings were available for 2 years prior to the COVID-19 lockdown and were obtained again some 10 days (range 9–13, s.d. 1.2) after official confinement. The authors certify that all procedures contributing to this study comply with the ethical standards of the relevant national and institutional committees on human experimentation and with the Helsinki Declaration of 1975, as revised in 2008. All procedures involving human participants or patients were approved by the *Unió Catalana d'Hospitals* ethics committee; approval numbers: CEIC 17/06 (phase 1), CEI 18/07 (phase 2). Written informed consent was obtained from all participants or patients. The data that support the findings of this study are available from the corresponding author, D.B.F., upon reasonable request.

**Fig. 1.**
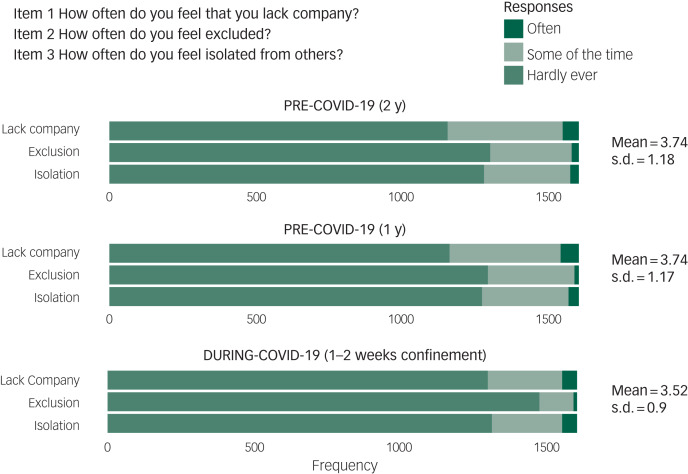
Frequency of loneliness responses before and during COVID-19 confinement.

The longitudinal three-item UCLA score (the sum of the three items of the scale) was modelled as a continuous variable using a linear mixed model allowing for a random intercept per participant. We also analysed each of the three individual items of the scale separately, after dichotomisation of the responses. As there were very few level 3 answers (‘often feeling lacking company, excluded, or isolated’: <4%, see Fig. 1), we collapsed them with level 2 (‘some of the times feeling …’) and opposed them to level 1 (‘hardly ever feeling …’). Thus, we obtained a separate binary longitudinal outcome for each of the three items of the scale, which we modelled with binomial linear mixed models including a random intercept per participant. Fixed effect parameters could be interpreted as odds ratios relative to the reference timepoint (2 years before the COVID-19 outbreak). Confidence intervals were estimated from model parameter standard deviations. All analyses were carried out with the functions lmer and glmer of the lme4 R package.^11^ Psychometric statistics were calculated following the standard formulas implemented in the psych R package.^12^

## Results

Ratings from the three UCLA items remained stable for the two pre-COVID-19 years (expected fixed effect intercepts: (2 years before) = 3.73, CI 3.70–3.75, (1 year before) = 3.73, CI 3.71–3.76). In clear contrast, scores fell significantly in the during COVID-19 assessment (3.52, CI 3.50–3.54) (see also descriptive statistics in Fig. 1).

Furthermore, this decrease did not occur homogeneously across all items of the UCLA scale. Instead, one item (feelings of being excluded from others, item 2) drove most of the reduction. Thus, the odds of ‘sometimes’ or ‘often’ feeling a lack of company (item 1), excluded from others (item 2) or isolated (item 3) presented reductions of varying degree, the largest emerging for feelings of being excluded (item 2). When the analysis was restricted to participants confined alone (*N* = 169), loneliness was overall higher than in those not confined alone before and during COVID-19: (2 years before) = 4.33, CI 4.15–4.53; (1 year before) = 4.28, CI 4.09–4.47 and (during COVID-19) = 4.14, CI 3.95–4.30. Investigating the UCLA items separately, we observed comparable patterns of significant reductions in loneliness during the confinement; once again, the largest reductions were observed in feelings of exclusion (Table 1).

**Table 1:** Odds ratios of loneliness items before and during COVID-19 confinement

	Odds ratio, hardly *v.* sometimes or often lonely	
	PRE (2 y)/PRE (1 y)	DURING/PRE (1 y)
All (*N* = 1604)		
Lack of company	1.02, CI 0.83–1.26	0.44, CI 0.36–0.55^a^
Exclusion	0.96, CI 0.78–1.21	0.23, CI 0.17–0.30^a^
Isolation	0.98, CI 0.80–1.2	0.79, CI 0.64–0.98^a^
Confined alone (*N* = 169)		
Lack of company	1.1, CI 0.60–2.03	0.47, CI 0.25, 0.86^a^
Exclusion	1.05, CI 0.56–1.96	0.20, CI 0.09–0.44^a^
Isolation	1.34, CI 0.79–2.27	1.39, CI 0.82–2.35

Odds ratios compare the odds of often or sometimes feeling lacking company, excluded or isolated compared with hardly ever in the two PRE-COVID-19 assessments (left column) and in the DURING-COVID-19 compared with the PRE-COVID-19 (1 y) assessment (right column). Top: odds obtained from all participants having answered the three loneliness assessments. Bottom: odds obtained from participants confined alone after COVID-19 outbreak and who already lived alone before the outbreak. Odds ratios are derived from a binomial linear mixed model with a random intercept for each participant; 95% confidence intervals are obtained from the estimated standard deviation of the regression coefficients.

a. 95% CI not including lack of effect (OR = 1).

The differences in the decreases in item score probabilities suggested a potential change in the internal coherence of the scale (cross-item correlation) after the pandemic outbreak. Indeed, we found that the DURING-COVID-19 pattern of scoring had altered the internal consistency of the UCLA scale. The two PRE-COVID-19 assessments exhibited good internal consistency (Cronbach's α = 0.76, CI 0.74–0.78, α = 0.75, CI 0.73–0.77) in agreement with the published literature,^10^ but in the DURING-COVID-19 evaluation, the consistency fell below psychometrically acceptable levels (α = 0.63, CI 0.60–0.66). The main driver of this disruption was again the change in scores for exclusion, its item-scale correlation dropping from *r* = 0.63 and 0.62 in the two PRE-COVID-19 assessments to *r* = 0.42 in the DURING-COVID-19 assessment.

## Discussion

Leveraging data from the BBHI study cohort, we observed that the influence of home confinement due to COVID-19 on the various subcomponents of loneliness presented differences, to the point of altering the established internal consistency of the UCLA scale. In other words, the subcomponents of the Loneliness scale were notably decoupled by the confinement. Feelings of exclusion from others (item 2) were the most reduced in all analyses, whereas scores on the Isolation subscale (item 3) decreased in the overall sample but were unchanged in people living alone. These findings support the notion that loneliness is not a unitary, isolated construct but rather represents a cluster of subjective and objective experiences of social integration and socioemotional states.^10^

The observation that the UCLA scale item ‘feelings of exclusion’ exhibited the greatest fall in score during a hard lockdown, which included an enforced reduction of physical social contacts, may be related to the general contextual component, namely, that people knew that there was nothing to be done about the limitation on social contacts, and that everybody else was in the same situation. This interpretation may be aligned with findings of a recent longitudinal study during COVID-19 lockdown conducted among university students who, during the confinement, reported worrying less about the possibility that others were having more rewarding experiences (i.e. fear of missing out) and perceived less competition.^6^ In our study, during lockdown, contact with close family members and friends may have been more easily maintained either within a shared household or using online methods, even though wider community life stopped, and this may have contributed to the decrease in feelings of exclusion.

In addition to this effect, there are other plausible sociological and psychological causes that might explain why feelings of exclusion suddenly dropped during the first weeks of lockdown in Spain. Some may appear obvious to many of us who lived through the experience, such as a sudden surge of patriotism, both spontaneous and fostered by the government's communication campaign, or a shift from a focus on personal problems towards a shared problem affecting the entire community. Our intriguing findings should invite replication with data from other countries, where strict lockdowns were imposed, or even building on social identity theory propounding that times of uncertainty and crisis predispose individuals to powerful identification with groups,^13^ and should encourage further research into the psychological and political predictors which, despite their observational nature, may shed light on certain psychological and sociopolitical causal mechanisms.

The main implications of our findings are that loneliness, and in particular feelings of inclusion and social belonging, can be improved without the need to enlarge the individual's physical social network. Therefore, programmes designed to address loneliness should not solely aim to ‘physically’ reconnect people (e.g., Shared Lives UK; see https://sharedlivesplus.org.uk/news-campaigns-and-jobs/growing-shared-lives/) but should consider incorporating or strengthening resources such as digital tools and the use of internet and social media to engage people creatively in new forms of social support, improving access to information about local community groups and promoting social integration. This is aligned with initiatives that aim to enhance collective creativity and increase the sense of ‘making things together’ which have been implemented during the quarantine in charitable campaigns such as the Campaign to End Loneliness (see https://bemoreus.org.uk/the-quarantine-quilt-connecting-through-creativity/).

Pending future assessments to determine whether feelings of loneliness fluctuate further during more extended periods of confinement or in the face of changes in the social context, the present findings provide new evidence of the psychological responses to a state of confinement, and may also offer valuable insights for future campaigns and interventions to reduce loneliness.^14^

## Data Availability

The data that support the findings of this study are available from the corresponding author, D.B.-F., upon reasonable request.
